# Akt2 Is Involved in Loss of Epithelial Cells and Renal Fibrosis following Unilateral Ureteral Obstruction

**DOI:** 10.1371/journal.pone.0105451

**Published:** 2014-08-22

**Authors:** Aiping Lan, Jing Zhang, Zhicheng Xiao, Xiaogang Peng, Yongfen Qi, Jie Du

**Affiliations:** Beijing An Zhen Hospital, Capital Medical University, The Key Laboratory of Remodeling-related Cardiovascular Diseases, Ministry of Education, Institute of Heart Lung and Blood Vessel Diseases, Beijing, China; Universidade de Sao Paulo, Brazil

## Abstract

Obstructive nephropathy is an aggressive form of chronic kidney disease (CKD), which is characterized by an epithelial-to-mesenchymal transition (EMT) and interstitial fibrosis. However, the molecular mechanisms of EMT and fibrosis are complex and not fully understood. In this study, we investigated the contribution of Akt2 to experimental renal EMT and fibrosis using the well-established model of unilateral ureteral obstruction (UUO). We found that Akt2 and phosphor (p)-Akt protein levels were increased in the obstructed kidneys. UUO induced activation of transforming growth factor-β1 (TGF-β1) signaling. Importantly, knockout of Akt2 suppressed UUO-induced EMT, kidney fibrosis, increased GSK3β activity, and decreased expression of Snail and β-catenin. Inhibition of GSK3β with LiCl (the inhibitor of GSK3β) increased the expression of Snail and β-catenin in cultured kidney epithelial cells. Our findings suggest that Akt2 partially contributes to interstitial fibrosis following UUO and that inhibition of this signaling pathway may provide a novel approach of prevent progression of renal fibrosis.

## Introduction

Renal interstitial fibrosis is the major pathological characteristic in progressive renal diseases, including nephropathy, eventually leading to end-point renal malfunction [1], [2]. The key feature of renal interstitial fibrosis is the accumulation and deposition of extracellular matrix (ECM), which is thought to be produced mainly by myofibroblasts [2], [3]. Over the past decade, Epithelial-mesenchymal transition (EMT) of tubular epithelial cells, characterized by loss of epithelial cell characteristics, and gain of ECM-producing myofibroblast characteristics, is an important pathway in myofibroblast production and is a key event in the pathogenesis and progression of renal interstitial fibrosis [4], [5], [6]. Recent cell lineage tracking experiments showed that EMT did not contribute to myofibroblast formation in kidney [7], [8]. These results suggest that EMT may not directly contribute to myofibroblast formation and fibrosis. However, loss of epithelial cells, namely EMT, may still contribute to myofibroblast formation and fibrosis indirectly as there are clear evidences of an EMT-like process occurs in renal epithelial cells [9]. For example, some studies have suggested that the loss of epithelial cells, or EMT, may indirectly contribute to interstitial fibrosis development through a paracrine signaling mechanism [9], [10]. Despite the novel nature of this hypothesis and increasing supportive evidence, the molecular mechanism of EMT and fibrosis has not been fully characterized.

It is reported that the Akt signaling has a critical role in mediating tubular EMT [11]. The Akt/PKB family of kinases, a downstream effector of phosphatidylinositol 3-kinase pathway, plays an important key role in regulating growth, proliferation, survival, metabolism, and other cellular activities [12], [13]. However, there are three major isoforms of Akt:Akt1, -2, and -3. Which one plays important role in the TGF-β1-induced EMT is not very clear. In previous in vitro study, we found that Akt2 activity is involved in TGF-β1-induced EMT in HK-2 cells, but whether Akt2 activity is involved in renal tubular EMT and renal fibrosis in vivo has not been reported.

Unilateral ureteral obstruction (UUO) in mice is a well-established experimental model resulting in tubulointerstitial fibrosis and tubular EMT in the obstructed kidney [14], [15]. Hence, in present study, we explored the role of Akt2 in renal tubular EMT and renal interstitial fibrosis following UUO, we found that Akt2 and phosphor(p)-Akt levels were increased in the obstructed kidneys, knockout (KO) of Akt2 suppressed UUO-induced EMT, kidney fibrosis. These results provide a new insight into the role of Akt2 in the UUO-induced kidney fibrosis and EMT.

## Materials and Methods

### Reagents and antibodies

Recombinant human TGF-β1 and LiCl were purchased from Sigma-Aldrich (St Louis, Mo, USA). The DMEM-F12 medium and fetal bovine serum (FBS), were supplied by Gibco (BRL Grand Island, NY, USA). Akt1, Akt2, Akt3, p-Akt (Thr308), p-Akt (Ser473), GSK3β (glycogen synthase kinase-3β), p-GSK3β, p-Smad3 and E-cadherin were purchased from Cell Signaling Technology (Beverly, MA). Collagen 1, α-SMA and Snail were obtained from Abcam (Cambridge, UK). TGF-β1, β-catenin, fibronectin, Vimentin and GAPDH were purchased from Santa Cruz Biotechnology (Santa Cruz, CA).

### Animals and Ethics Statement

Akt2 knockout mice and wild-type littermates were purchased from the Jackson Laboratory. Mice were maintained under specific-pathogen-free conditions in the animal facility at the Beijing Heart Lung and Blood Vessel Diseases Institute. The mice were given a standard rodent chow and water ad libitum. All animal care and experimental protocols complied with the US National Institutes of Health Guide for the Care and Use of Laboratory Animals (publication no. 85-23, 1996) and were approved by the Institutional Animal Care and Use Committee of Capital Medical University.

### UUO model in mice

Surgical procedures were performed in 2-month-old mice as we previously described [16] with minor modification. Six WT littermate and Akt2 KO mice (in C57/BL6 genetic background), of 20–30 g body weight, in each group were studied, UUO was carried out as described [16]. Mice were anesthetized with pentobarbital, the abdomen was opened, and the left ureter was ligated with 5-0 silk. Then the abdomen was closed with running sutures and the skin was closed with interrupted sutures. Sham-operated mice had their ureter exposed but not ligated. After surgery, the mice were maintained in a temperature-controlled room.

### Cell culture and Treatments

HK-2 cells were grown in DMEM-F12 medium supplemented with 10% FBS, 2 mmol/L 1-glutamine, 100 U/ml penicillin, and 100 µg/ml streptomycin at 37°C under an atmosphere of 5% CO_2_ and 95% air. For TGF-β1 treatments, HK-2 cells (2×10^5^) cultured in six-well plates were made quiescent by culturing in medium containing 0.1% FBS for 24 h before treatment with10 ng/ml TGF-β1 and 40 mmol/L LiCl.

### Histopathology and Immunohistochemistry

After 3 days, 5 days, and 7 days of surgery, mice were enthanized by an overdose of pentotarital (100 mg/kg). Renal morphology was examined in 10% neutral-buffered formalin-fixed, paraffin-embedded tissue sections after they were stained with Masson's-modified trichrome. For immunohistochemical staining assay, kidneys were perfused as described [16]. After removing paraffin, sections were incubated for 30 min in 3% hydrogen peroxide (H_2_O_2_) in methanol at room temperature (RT), washed with PBS, and heated in a microwave in 10 mM citrate buffer (pH 6.0) for 20 min. Sections were blocked using 10% goat serum (Vector Laboratories, Burlingame, CA, USA) for 30 min, incubated with anti-fibronectin, anti-Snail, anti-β-catenin, anti-p-Akt (Ser473), anti-p-GSK3β, anti-E-cadherin, anti-α-SMA (1∶200 dilution). The staining protocol was performed according to the ABC kits (Vector Lab). The signals were visualized using a peroxidase substrate kit (Vector Lab). The pictures were recorded using a Nikon light microscope.

### Western blot analysis

Total cells and tissues were homogenized in lysis buffer and centrifuged at 14,000 g for 30 min. Supernatant was recovered and proteins were quantified using the BCA protein assay kit. Lysates (60 µg per lane for tissue, 30 µg per lane for cells) were loaded onto 12% SDS–polyacrylamide gel electrophoresis (PAGE), and the proteins were transferred to polyvinylidene difluoride (PVDF) membranes. Membranes were blocked for 1.5 h at room time (RT) in fresh blocking buffer (0.1% Tween20 in Tris-buffered saline (TBS-T) containing 5% fat-free milk) and then incubated with the primary antibodies anti-p-Akt (Ser473), anti-p-Akt (Thr 308), anti-Akt1, anti-Akt2, anti-Akt3, anti-TGFβ1, anti-p-Smad3, anti-vimentin, anti-E-cadherin, anti-α-SMA, anti-Snail, anti-β-catenin, anti-p-GSK3β, anti-t-GSK3β, anti-fibronectin, anti-collagen 1(1∶1000 dilution), or GAPDH antibodies, overnight at 4°C, then secondary antibodies (1∶5000 dilution, horseradish peroxidase-conjugated goat anti-rabbit IgG) for 1.5 h at RT. Membranes were washed three times with TBS-T and the signals were visualized and analyzed using Odyssey image system (Li-COR, Lincoln, NE, USA). Each experiment was repeated at least three times. The density of specific bands was measured and normalized to that of GAPDH.

### Statistics

All data are representative of experiments done in triplicate and are expressed as the means ± SD. Differences between groups were analyzed by one-way analysis of variance (ANOVA) using SPSS 13.0 software. A *P*<0.05 was considered statistically significant.

## Results

### Akt2 deficiency decreases UUO-induced fibrosis

**Figure 1:**
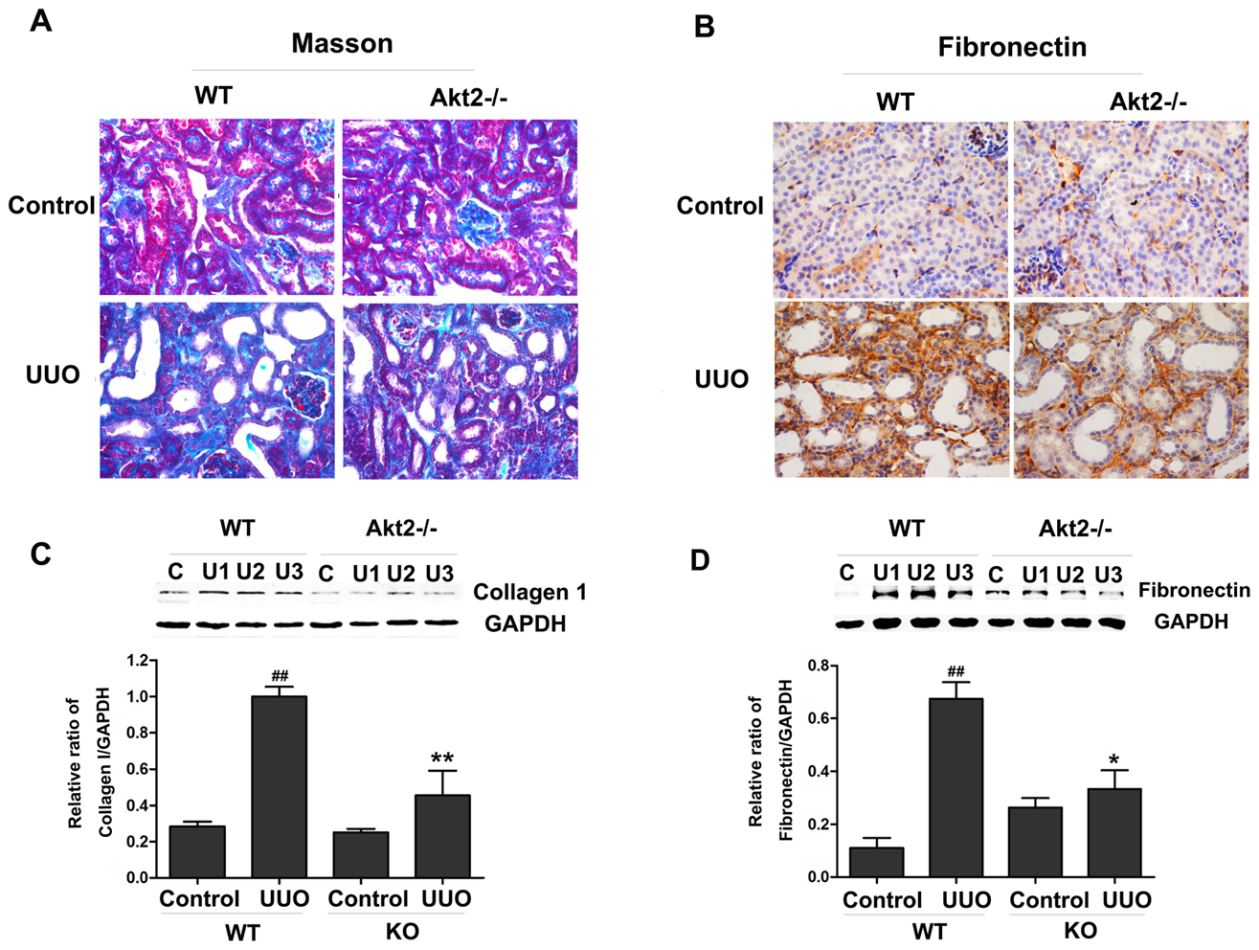
Unilateral ureteral obstruction (UUO)-induced kidney fibrosis was attenuated in Akt2 knockout (KO) mice. (A) Sections from control and obstructed kidneys from WT and Akt2 KO mice were stained with Masson's-modified trichrome. The blue color shows extracellular collagen deposition. (B) Immunohistochemistry for Fibronectin in unobstructed and obstructed kidneys from WT and Akt2 KO mice. Magnification: ×400. (C and D) Western blot analysis of Fibronectin and Collagen I protein expression in obstructed and unobstructed kidneys of WT and Akt2 KO mice. GAPDH was used as internal loading control. Band intensities were calculated using Scion Image software. Values are means ± SD (n = 6). ^##^ *P*<0.01 compared to the. nonobstructive kidney from WT mice; ^*^ *P*<0.05, ^**^ *P*<0.01 compared to the obstructive kidney from WT mice.

After 7 days of surgery, the obstructive kidney in WT mice showed the typical features of obstructive nephropathy: tubular dilatation and widespread renal tubulointerstitial damage and fibrosis, evidenced by the increase in collagen deposition as assessed by Masson's-modified trichrome staining (Figure 1A) and western blot (Figure 1C). In contrast, kidneys from Akt2 KO mice were protected against UUO-induced collagen deposition (Figure 1 A and C). In addition, the effects of UUO on the levels of fibronectin protein expression were significantly reduced in Akt2 KO mice by western blot assay and immunohistochemistry (Figure 1 B and D). Although we found the level of fibronectin protein in the unobstructed kidneys from Akt2 KO mice is more than that in the unobstructed kidneys from WT mice, we speculated this due to individual variation. All the above results reveal that Akt2 depletion attenuates UUO-induced fibrosis.

### Akt2 and Active Akt levels are increased in the obstructed kidneys

**Figure 2:**
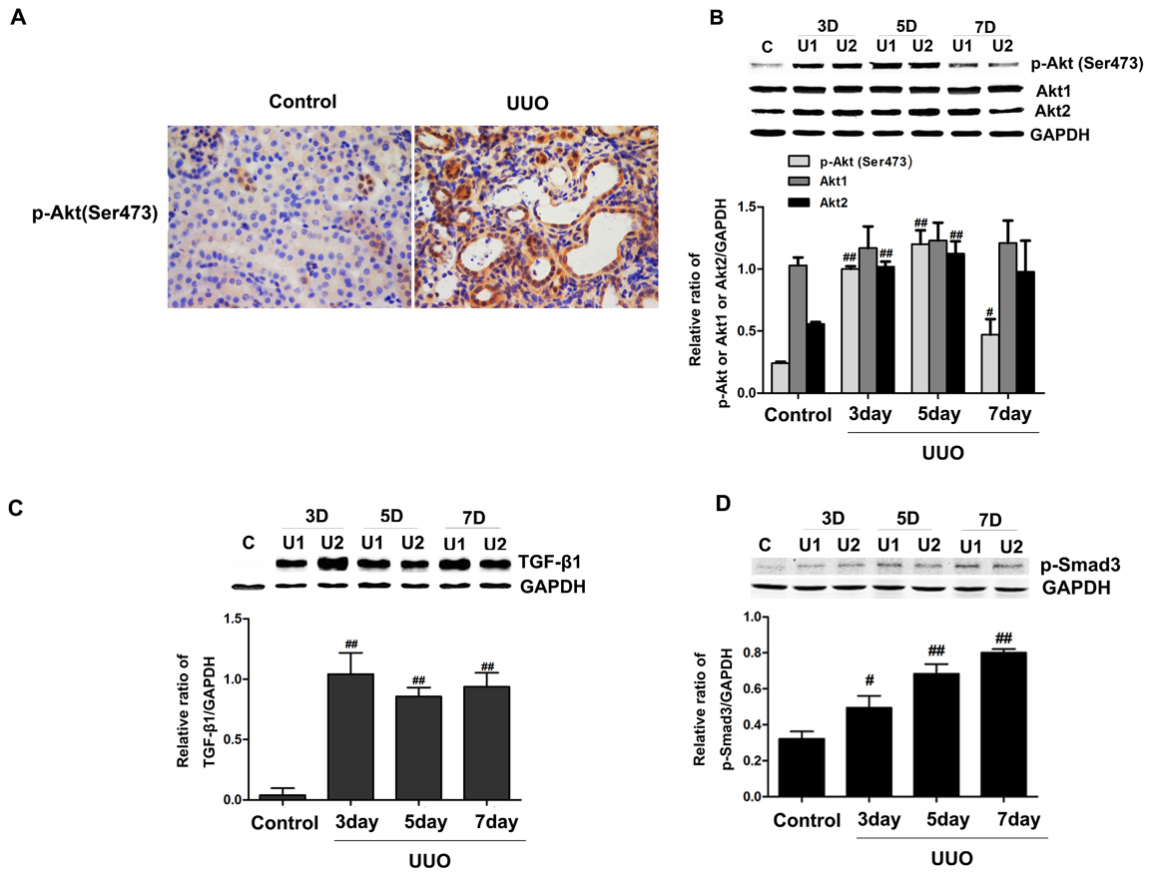
Unilateral ureteral obstruction (UUO) activated Akt and TGF-β1 signaling in obstructed kidneys. (A) Immunohistochemical staining of p-Akt(Ser473) in wide-type (WT) mice after surgery for 5 days. Magnification: ×400. (B) Time course for the effects of UUO on expression of p-Akt(Ser473), Akt1 and Akt2, respectively by western blot analysis. (C and D) Time course for the effects of UUO on expression of TGFβ1 and p-Smad3 in mouse kidneys by western blot analysis. GAPDH was used as internal loading control. Band intensities were calculated using Scion Image software. Data are presented as means ± SD (n = 6). ^#^ *P*<0.05, ^##^ *P*<0.01 compared to the control group.

We examined active Akt levels in the obstructed kidneys from WT mice. As shown in Figure 2B, Western blot demonstrated similar results: p-Akt (Ser473) protein was significantly elevated in the obstructive kidneys as compared with that in the unobstructed, contra-lateral kidneys. This data was confirmed by immunohistochemical analysis as shown in Figure 2A, increased levels of p-Akt (Ser473) was present in obstructed kidneys compared with control kidneys, with staining predominantly located in the kidney tubules and interstitium. We also examined the expression of p-Akt (Thr308) protein in obstructed kidneys, we found that the level of p-Akt (Thr308) protein is not significantly increased following UUO (Fig. S1). It is known that Akt1 and Akt2 but not Akt3 mRNA is expressed in the kidneys [17]. Next, we examined the expression of Akt1 protein and Akt2 protein in the obstructive kidneys. Western blot analysis showed that Akt2 protein levels were elevated in the obstructed kidneys compared with that in the unobstructed kidneys from WT mice, whereas Akt1 protein levels were similar among groups (Figure 2B). Thus Akt2 expression is enhanced in the obstructive kidneys, and that Akt signaling in obstructive kidneys may have been activated.

We also detected increases in protein levels of TGF-β1 and phosphor (p)-Smad3 in obstructed kidneys compared with control kidneys (Figure 2 C and D), suggesting that amplified TGF-β1 signaling was presented in obstructed kidneys. As TGF-β1 is a key inducer of EMT and fibrosis [18], we hypothesized that elevated levels of TGF-β1 would have consequences for EMT and fibrosis in the renal tubules.

### Akt2 deficiency suppresses UUO-induced EMT

**Figure 3:**
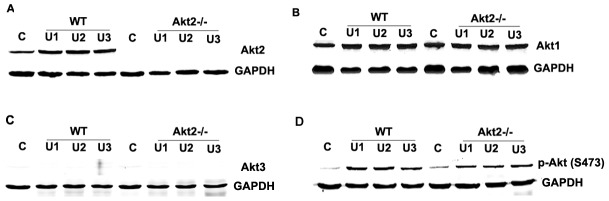
Akt isoforms and p-Akt (Ser473) protein expressions in the kidneys from wild type (WT) and Akt2 knockout (KO) mice. Akt2, Akt1, Akt3 and p-Akt(Ser473) protein expression in the obstructed kidneys isolated from WT and Akt2 KO mice 5 day after UUO were detected by the western blotting. (A) The expression levels of Akt2 protein in WT mice and Akt2 KO mice by western blotting analysis. (B) The expression levels of Akt1 in WT mice and Akt2 KO mice by western blotting analysis. (C) The expression levels of Akt3 protein in WT mice and Akt2 KO mice by western blotting analysis. (D) The expression levels of p-Akt (Ser473) protein in WT mice and Akt2 KO mice by western blotting analysis. GAPDH was used as internal loading control.

To further determine whether Akt2 contributes to EMT following UUO, we firstly examined the expression of Akt1 protein and Akt2 protein in the obstructed kidneys from WT mice or Akt2 knockout (KO) mice. As shown in Figure 3 A and B, the protein expression of Akt1 were presented in both WT mice and Akt2 KO mice, but the expression of Akt2 was presented in only WT mice, indicating that the expression of Akt1 is not impacted in Akt2 KO mice and Akt2 KO is specific. In order to see whether there is a complementary expression of Akt3 in Akt2 KO mice, we also test the expression of Akt3, as shown in Figure 3 C, there is no Akt3 expression in kidneys of WT and Akt2 KO mice. In addition, we detected that p-Akt (Ser473) expression is also increased in Akt2 KO mice, which is less than that in WT kidneys (Figure 3 D), this may explain the partially influence of Akt2 KO on the fibrosis following UUO.

**Figure 4:**
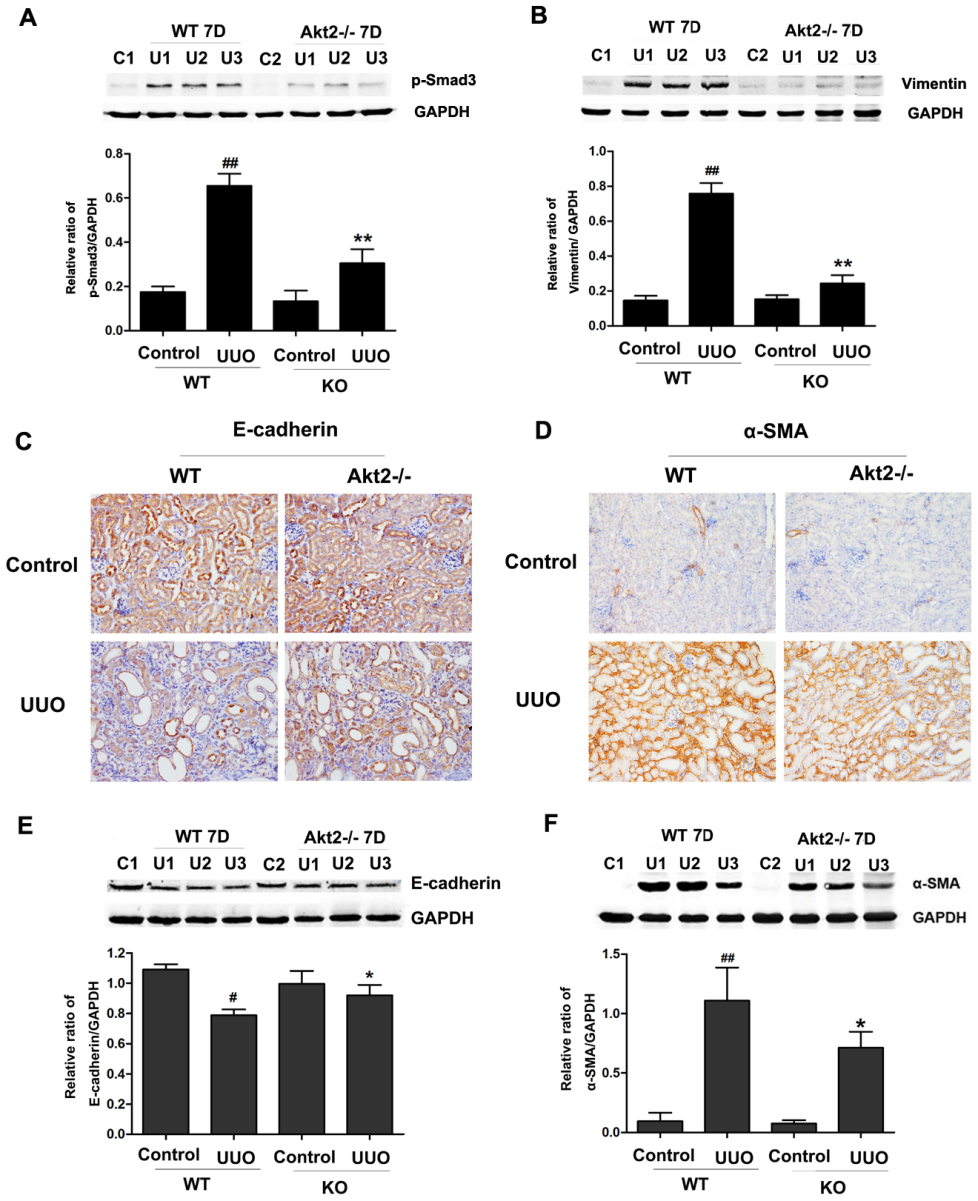
Unilateral ureteral obstruction (UUO)-stimulated epithelial-mesenchymal transition (EMT) was blocked by Akt2 knockout (KO). (A and B) Western blot analysis of p-Smad3 and vimentin protein expression in unobstructed and obstructed kidneys from WT and Akt2 KO mice. (C and D) Immunohistochemistry for E-cadherin and α-smooth-muscle actin (α-SMA) in unobstructed and obstructed kidneys from WT and Akt2 KO mice. Magnification: ×200. (E and F) Western blot analysis of E-cadherin and α-SMA protein expression in unobstructed and obstructed kidneys from WT and Akt2 KO mice. GAPDH was used as internal loading control. Band intensities were calculated using Scion Image software. Data are shown as mean ± SD (n = 6). ^#^ *P*<0.05, ^##^ *P*<0.01 compared to the nonobstructive kidney from WT mice; ^*^ *P*<0.05 compared to the obstructive kidneys from WT mice.

As the TGF-β1/Smad3 signaling is the classical pathway in EMT, so we investigated the effect of Akt2 KO on the expression of p-Smad3, as shown in Figure 4 A, the expression level of p-Smad3 protein was markedly increased as compared with that in unobstructed kidneys of WT mice, p-Smad3 expression in the obstructed kidneys of Akt2 KO mice was less as compared with kidneys from obstructed WT mice, suggesting that Akt2 KO may affect EMT following UUO. Next, we measured protein expression of EMT markers vimentin (Figure 4 B) and E-cadherin (Figure 4 C, and E) using western blotting and immunohistochemical staining. As shown in Figure 4 B, C and E, after 7 days of surgery, the expression level of vimentin protein was markedly increased and the expression level of E-cadherin protein was significantly decreased as compared with that in unobstructed kidneys of WT mice. However, Increased vimentin expression and decreased E-cadherin expression were less in the obstructed kidneys of Akt2 KO mice (Figure 4 B, C and E), indicating that Akt2 KO could affect EMT following UUO. Next, we measured the expression of α-smooth-muscle actin (α-SMA) in the kidneys of WT and Akt2 KO mice, as shown in Figure 4 D and F, the expression level of α-SMA protein was markedly increased as compared with that in unobstructed kidneys of WT mice, but this increase is lower in Akt2 KO mice than in WT mice (Figure 4 D and F). These data suggest that UUO-induced EMT and fibrosis is suppressed in Akt2 KO mice.

### Akt2 depletion suppresses UUO-induced the expression of Snail and β-catenin in the obstructed kidneys

**Figure 5:**
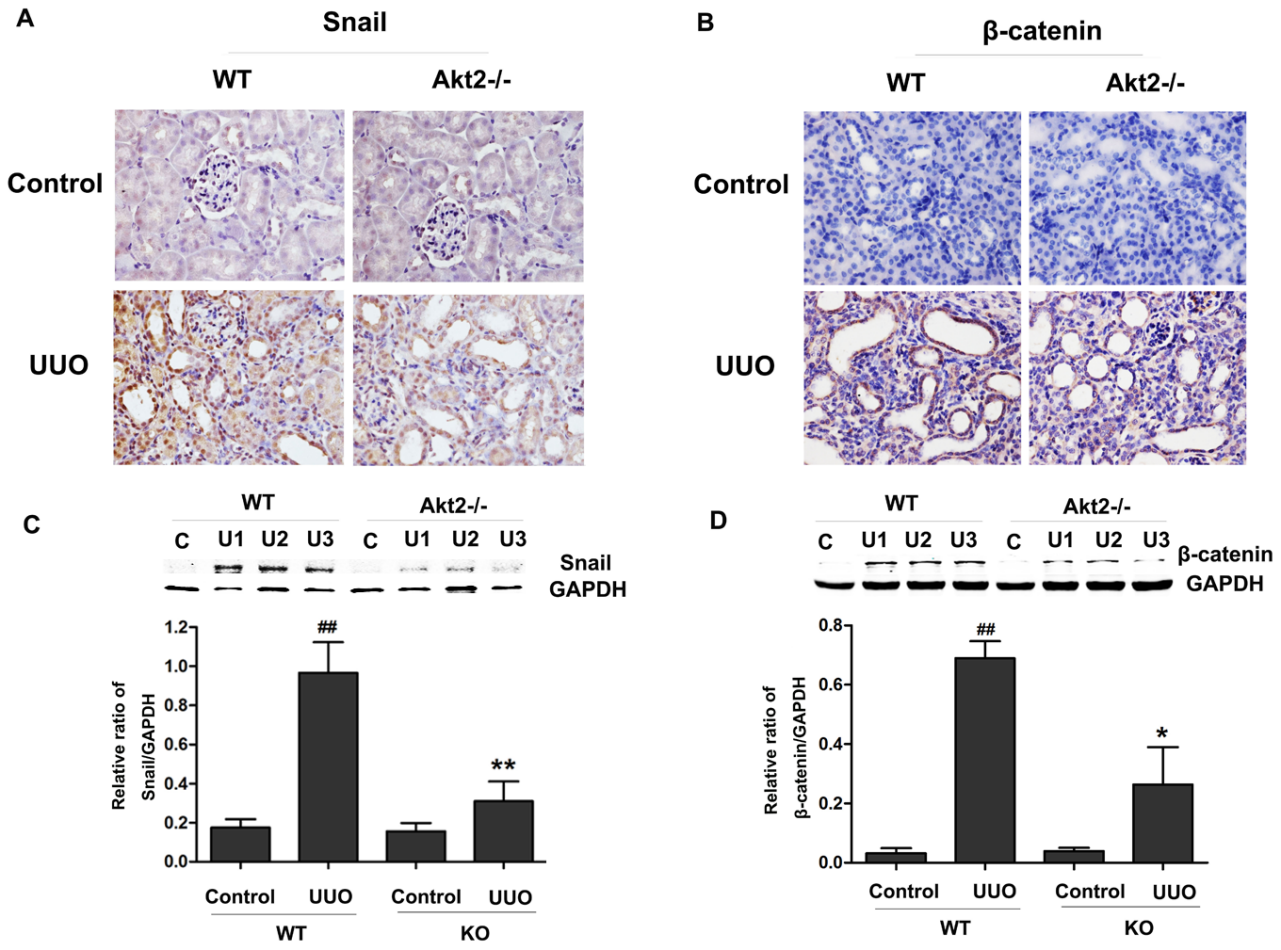
UUO-induced expression of Snail and β-catenin was suppressed by Akt2 knockout (KO) in vivo. (A and B) Immunohistichemistry for Snail and β-catenin in unobstructed and obstructed kidneys from WT and Akt2 KO mice. Magnification ×400. (C and D) Western blot analysis of Snail and β-catenin protein expression in unobstructed and obstructed kidneys from WT and Akt2 KO mice. GAPDH was used as internal loading control. Band intensities were calculated using Scion Image software. Data are expressed as mean ± SD (n = 6). ^##^ *P*<0.01 compared to the nonobstructive kidney from WT mice; ^*^ *P*<0.05 compared to the obstructive kidney from WT mice.

Snail is a key transcriptional repressor of E-cadherin, contributes to EMT [19], we therefore assessed the expression of Snail in UUO kidneys. As shown in Figure 5 A and C, after 7 days of surgery, the expression level of Snail protein was increased as compared with that in unobstructed kidneys of WT mice. However, Snail expression in the obstructed kidneys of Akt2 KO mice was less increased as compared with kidneys from obstructed WT mice. Something similar occurs with β-catenin expression, which increased significantly after UUO in WT mice, but not in Akt2 KO mice (Figure 5 B and D). The above findings suggest that Akt2 partially mediates the expression of Snail and β-catenin induced by UUO.

### UUO activated Akt2 promotes the expression of Snail and β-catenin depends on GSK3β deactivation in the obstructive kidneys

**Figure 6:**
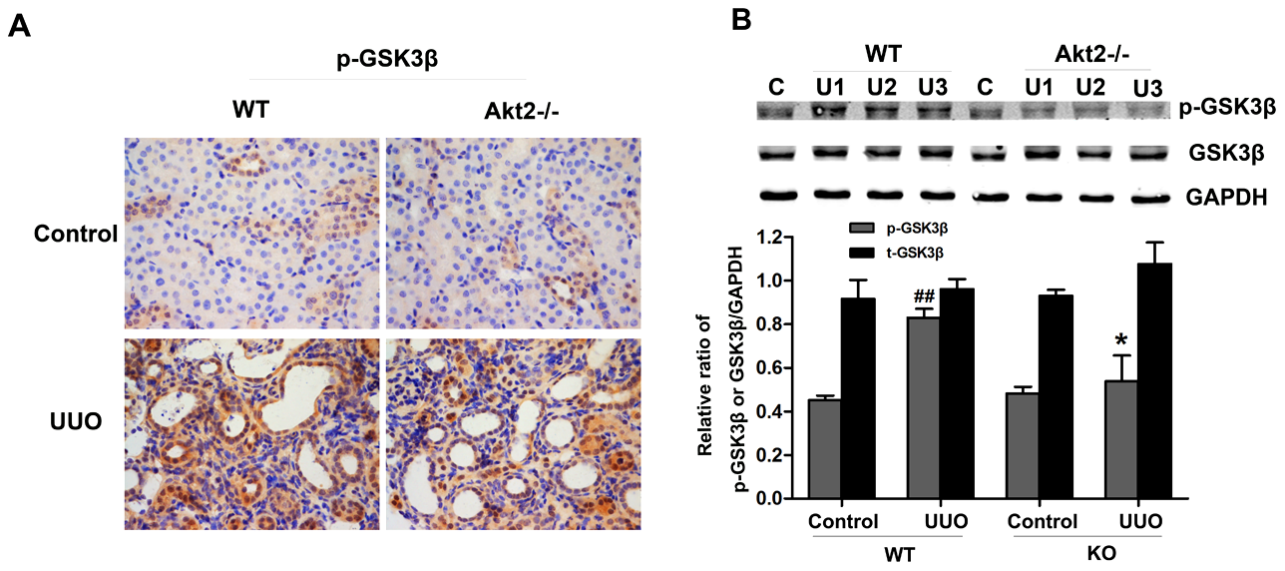
Effects of Akt2 deficiency on the phosphorylation of GSK3β after UUO. (A) Immunohistichemistry for p-GSK3β in unobstructed and obstructed kidneys from WT and Akt2 KO mice. Magnification: ×400. (B) Western blot analysis of p-GSK3β and t-GSK3β protein expression in unobstructed and obstructed kidneys from WT and Akt2 KO mice. GAPDH was used as internal loading control. Band intensities were calculated using Scion Image software. Data are presented as mean ±SD (n = 6). ^##^ *P*<0.01 compared to the nonobstructive kidney from WT mice; ^*^ *P*<0.05 compared to the obstructive kidney from WT mice.

To determine how activated Akt2 promotes expression of Snail and β-catenin following UUO. We examined the effect of Akt2 KO on the expression of p-GSK3β. GSK3β, as a downstream target of PI3K and Wnt pathways, is necessary for the maintenance of epithelial architecture [20]. As shown in Figure 6, after 7 days of surgery, the expression of phosphor (p) GSK3β was increased as compared with that in unobstructed kidneys of WT mice, which is suppressed in Akt2 KO obstructed kidneys.

**Figure 7:**
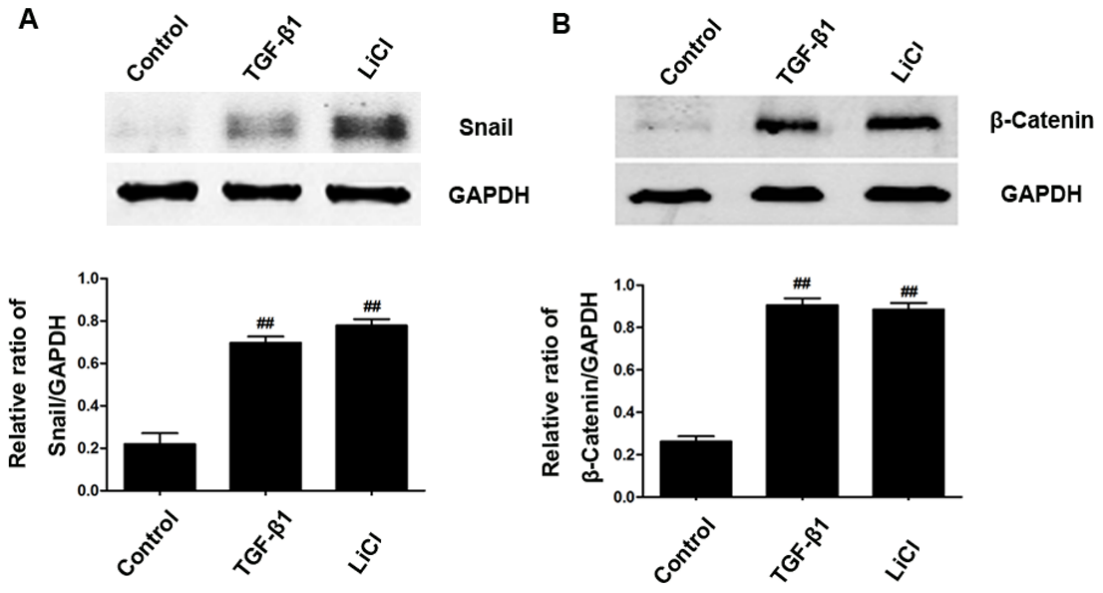
Deactivation of GSK3β mediated activation of Snail and β-catenin in tubular epithelial cells. (A and B) Cells were treated with TGF-β or GSK3 inhibitor (LiCl 40 mmol/L). Prptein was then extracted and blotted with antibodies against Snail and β-Catenin. GAPDH was used as internal loading control. Band intensities were calculated using Scion Image software. Data are shown as mean ± SD (n = 3). ^##^ *P*<0.01 compared to the control group; ^**^ *P*<0.01 compared to the TGF-β1 treated group.

In order to examine the role of GSK3β in the expression of Snail and β-catenin in HK-2 cells, we used TGF-β1 or LiCl to treat cells. As shown in Figure 7, TGF-β1-induced expression of Snail and β-catenin (Figure 7). Inhibition of GSK3β with LiCl_2_ (the inhibitor of GSK3β) also increased the expression of Snail and β-catenin (Figure 7), indicating that GSK3β deactivation is involed in expression of Snail and β-catenin in the tubule epithelial cells.

## Discussion

In this study, we reported that UUO induced lower interstitial fibrosis, in Akt2 KO than in WT mice. All the fibrogenic hallmarks of renal fibrosis were attenuated in the obstructed kidneys from the Akt2 KO mice, including the less of fibronectin and collagen I accumulation. Indicating that Akt2 is involved in the development of renal fibrosis. EMT is thought to be a significant component of renal fibrosis. We found the levels of TGF-β1 and p-Smad3 protein were both increased in obstructed kidneys (Figure 2 C and D). These data suggested that amplified TGF-β signaling was present in kidneys, which would have consequences for epithelial cells integrity in the renal tubules. Furthermore, we also found that p-Akt (Ser473) was increased in UUO (Figure 2 A and B). Indicating us that Akt activation may be elicited by TGF-β1 and involved in loss of tubular epithelial cells in UUO. Firstly, we found p-Smad3 expression in the obstructed kidneys of Akt2 KO mice was less as compared with kidneys from obstructed WT mice, suggesting that Akt2 KO may affect EMT following UUO. Secondly, the level of vimentin, E-cadherin and α-SMA was detected and we found that Akt2 KO suppressed increases in vimentin and α-SMA, while loss of E-cadherin was prevented by Akt2 KO (Figure 4). Although Akt2 KO abolished the UUO-induced increase in vimentin and α-SMA, UUO-induced alteration in E-cadherin were only partially recovered, suggesting that Akt2 signaling is not the only pathway involved in the EMT following UUO.

The present observations further provide insight into the Akt2-dependent mechanisms leading to EMT in vivo. Snail, a zinc finger transcription factor, has been characterized as a key regulator EMT. Many studies have shown that Snail binds to specific DNA sequences called E-boxes in the promoter of the E-cadherin gene to repress transcription of E-cadherin [16]. E-cadherin is a major gene in the epithelium, which is an important determinant in maintaining the epithelial phenotype [21]. In our study, we found that UUO increased Snail accumulation of kidney tubule cells (Figure 5A), and the influence of UUO on the expression of Snail was at least partially mediated by Akt2, as the expression of Snail after UUO was blocked in kidneys of Akt2 KO mice (Figure 5 A and C). These results are consistent with our vitro data that TGF-β1 induced the expression of Snail, the effect of TGF-β1 on Snail expression was sensitive to the presence of Akt2 in HK-2 cells. Furthermore, Villagrasa P, et al. reports that Akt2 interacts with Snail on its target promoters and regulates transcription [22], which is consistent with our findings. β-catenin signal cascade, a principal mediator of canonical Wnt signaling, plays a fundamental role in regulating various biologic processes such as organ development, tissue homeostasis, and pathogenesis of human disease [23], [24]. It is reported that β-catenin signaling is activated in a wide variety of fibrotic CKDs such as obstructive nephropathy. Consistent with previous studies [16], we also found that a marked increase of β-catenin protein in WT obstructed kidneys, which was blocked in Akt2 KO obstructed kidneys (Figure 5 B and D). Our findings are supported by a recent evidence that elevated Akt2 levels in the Irs2-/- kidney lead to increased GSK3β phosphorylation and increased β-catenin levels [25] In addition to Wnt, β-catenin activation is also regulated by other signal pathways such as TGF-β1. In cultured HK-2 cells, we observed that TGF-β1-induced β-catenin activation (Figure 7B).

To determine how activated Akt2 promotes expression of Snail and β-catenin following UUO. We examined the effect of Akt2 KO on the expression of p-GSK3β. GSK3β, as an active kinase of Snail, can bind to Snail and facilitate its proteasomal degradation [26]. In addition, GSK3β, as a downstream target of the PI3K/Akt and Wnt pathways, is necessary for the maintenance of epithelial architecture [27]. According to our observations, UUO increased GSK3β phosphorylation in kidneys (Figure 6), which was attenuated in obstructed Akt2 KO mice. Our findings are consistent with the previous studies [25]. Importantly, inhibiting GSK3β with LiCl increased Snail and β-catenin expression in HK-2 cells. The above results indicated that Akt2 partially mediated UUO-induced Snail and β-catenin expression, but it rather effective owing to its influence of GSK3β.

Taken together our results show that, after UUO, the kidneys of Akt2 KO mice showed a lower interstitial fibrosis and EMT than those of WT animals due to a increase of GSK3β activity and a decrease of Snail and β-catenin expression. Our data show a direct role of the Akt2 signaling pathway in the development of renal tubule EMT and fibrosis and point to its inhibitors as potential antifibrotic drugs.

## Supporting Information

Figure S1 **Time course for the effects of UUO on expression of p-Akt (Thr 308).**
(DOCX)
