# Supplementary figures and images for: Akt2 Is Involved in Loss of Epithelial Cells and Renal Fibrosis following Unilateral Ureteral Obstruction
Source: PLoS One. 2014 Aug 22;9(8):e105451. doi: 10.1371/journal.pone.0105451 (PMC4141797; doi:10.1371/journal.pone.0105451)

**Supplemental Figure 1 Time course for the effects of UUO on expression of p-Akt (Thr 308).**


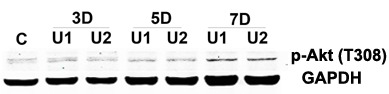

Supplement: Figure S1 — Time course for the effects of UUO on expression of p-Akt (Thr 308). (DOCX) [file pone.0105451.s001.docx]
